# Supplementary material for: Forming cognitive maps for abstract spaces: the roles of the human hippocampus and orbitofrontal cortex
Source: Commun Biol. 2024 May 1;7:517. doi: 10.1038/s42003-024-06214-5 (PMC11063219; doi:10.1038/s42003-024-06214-5)
Supplement: Supplementary file 2 — Supplementary Information [file 42003_2024_6214_MOESM2_ESM.pdf]

# Forming cognitive maps for abstract spaces: the roles of the human hippocampus and orbitofrontal cortex

Yidan Qiu[1], Huakang Li[2], Jiajun Liao[1], Kemeng Chen[1], Xiaoyan Wu[1],

Bingyi Liu[1], Ruiwang Huang[1*]

[1] School of Psychology; Center for the Study of Applied Psychology; Key Laboratory of Mental Health and Cognitive Science of Guangdong Province; Key Laboratory of Brain, Cognition and Education Sciences, Ministry of Education; South China Normal University, Guangzhou 510631, China.

[2] School of Computer Science and Engineering, South China University of Technology, Guangzhou, 510006, China.

* Correspondence should be addressed to

Ruiwang Huang, PhD
School of Psychology
South China Normal University
Guangzhou 510631, China
Tel/Fax: +86 (0)20-8521 6499

Email: ruiwang.huang@gmail.com

## Supplementary Information

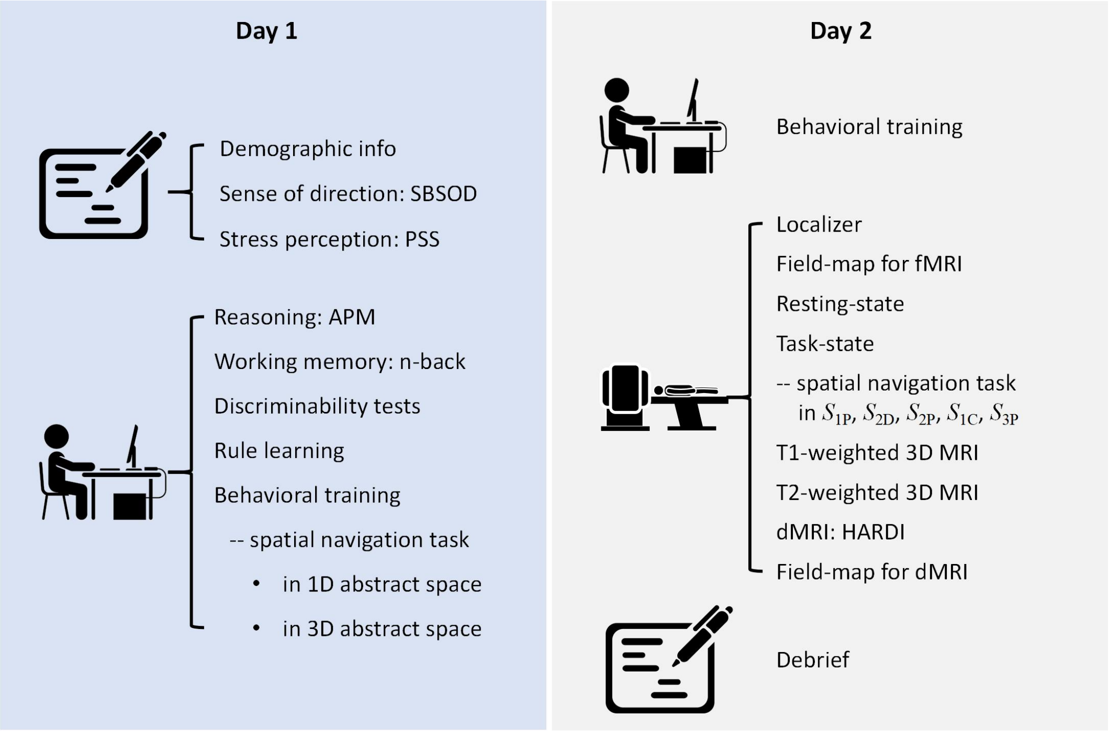


**Fig. S1.** Procedure of the experiment in two days. On Day 1, the subjects filled out three questionnaires and completed five cognitive tests. On Day 2, the subjects first completed a behavioral training task and then attended the MRI scanning. Afterwards, we asked the subjects about their strategy used in the navigation task. The symbols are sourced from the WPS Docer database (https://docer.wps.cn/) and are non-commercial free materials for personal communication and learning purposes, as per the authorization policy. Abbreviations: info, information; SBSOD, Santa Barbara sense of direction scale; PSS, Perceived Stress Scale; APM, Raven's advanced progressive matrices; $S_{1P}$, $S_{1C}$, $S_{2P}$, $S_{2C}$, and $S_{3P}$ indicate the five abstract spaces; dMRI, diffusion-weight MRI; HARDI, high angular resolution diffusion-weighted imaging.

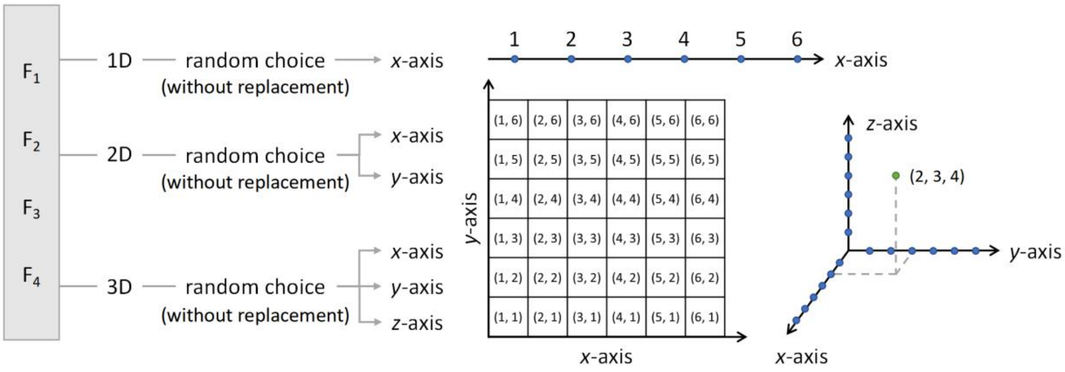


**Fig. S2.** Schematic diagram of the coordinate assignment to the locations in an abstract space. The chosen dimensions (features of the basic symbols shown in Fig. 1) were assigned as the *x*-, *y*-, and *z*-axes, respectively. The coordinate of a location was represented as (*x*) in a 1D space, (*x*, *y*) in a 2D space, and (*x*, *y*, *z*) in a 3D space. For example, the coordinate of the first location was (1) in 1D space, (1, 1) in 2D space, and (1, 1, 1) in 3D space.

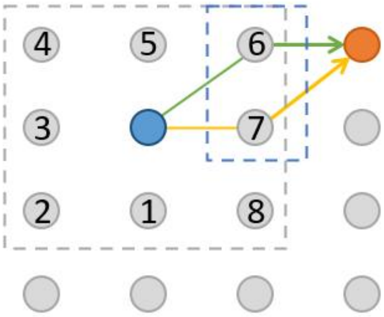


**Fig. S3.** An example navigational path in a 2D abstract space. The circle in blue indicates the current location, the circle in orange indicates the destination, and the green and yellow lines are the shortest paths from the current location to the destination. The numbers in the circle indicate the order that the selected options would be sorted.

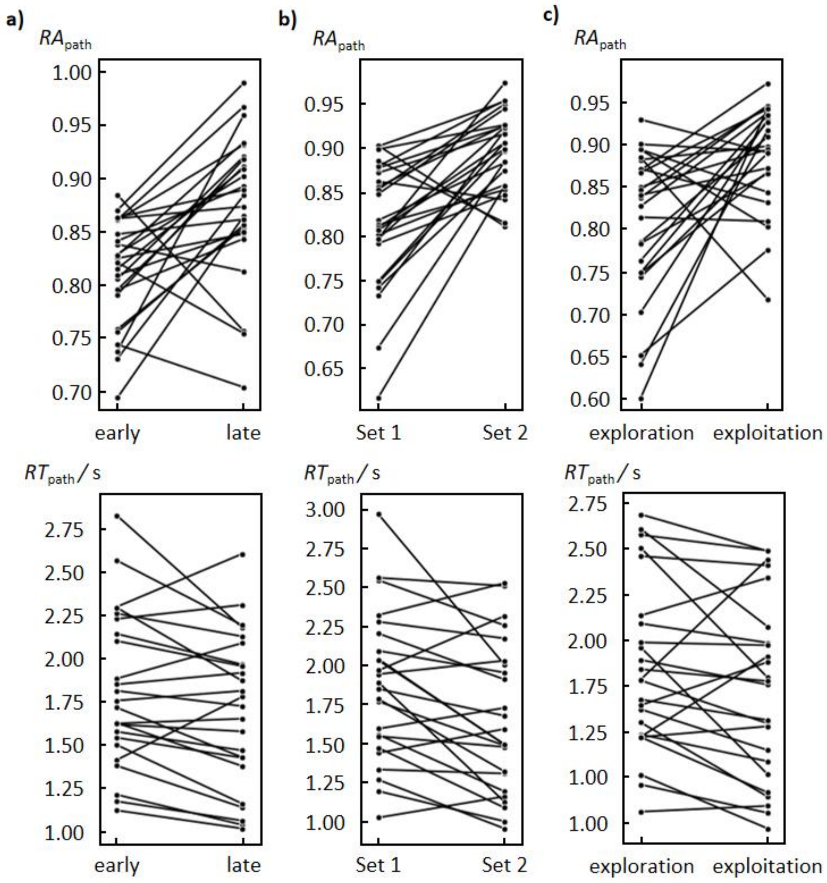


**Fig. S4.** Behavioral performance corresponding to the three LMM analyses. **(a)** The values of $RA_{path} = \sum_{i=1}^{N_{step}} (RA_i) / N_{step}, i \in (1, 2, ..., N_{step})$ and $RT_{path} = \sum_{i=1}^{N_{step}} (RT_i) / N_{step}$ in the early learning and the late learning phases. **(b)** The values of $RA_{\mathrm{path}}$ and $RT_{\mathrm{path}}$ of the first (Set 1) and the second space (Set 2) of the 1D and 2D spaces. **(c)** The $RA_{\mathrm{path}}$ and $RT_{\mathrm{path}}$ of the exploration and exploitation stages. Each dot corresponds to a subject, and the lines connect the data from the same subject.

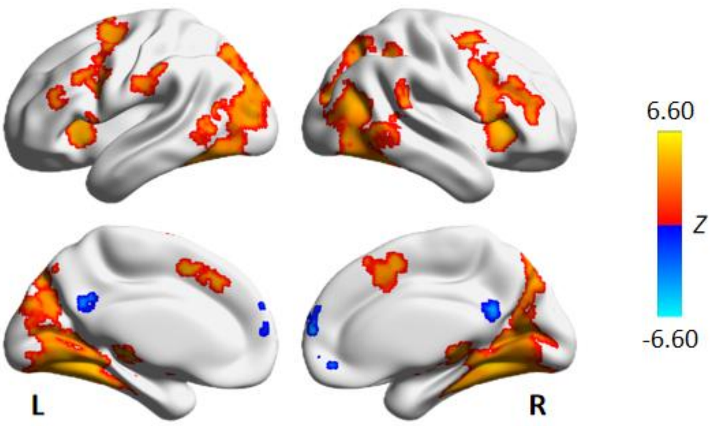


**Fig. S5.** Brain activation significantly associative with the learning level. Regions with the warm (cold) colors for positive (negative) association with the learning level. The color bar indicates the range of *Z*-values. The underlying statistical maps are available at https://identifiers.org/neurovault.collection:16948.

**Table S1.** Behavioral performances of the subjects in the navigation task during the fMRI scanning in the five abstract spaces.

| **Space** | **# Data length (*SD*)** | **RA (*SD*)** | **RT (*SD*, s)** | **# Trial (*SD*)** |
|---|---|---|---|---|
| $S_{1P}$ | 238.84 (42.35) | 0.92 (0.27) | 1.05 (0.51) | 2.05 (1.20) |
| $S_{1C}$ | 242.64 (43.89) | 0.95 (0.21) | 1.20 (0.94) | 2.07 (1.20) |
| $S_{2C}$ | 374.48 (97.60) | 0.64 (0.48) | 2.25 (1.57) | 3.24 (2.19) |
| $S_{2P}$ | 382.76 (61.19) | 0.75 (0.43) | 2.54 (1.67) | 3.23 (2.14) |
| $S_{3P}$ | 427.32 (56.52) | 0.71 (0.46) | 2.73 (1.87) | 3.66 (2.06) |

*Notes:* Data length refers to the mean number of brain volumes acquired in the fMRI scan across all subjects, with the repetition time TR = 1,500 ms. *SD* stands for the standard deviation. The number of paths was the mean number of paths across all subjects. The response accuracy (RA) and response time (RT) were calculated by averaging the response accuracy of the paths ($RA_{\text{path}}$) and the response time of the paths ($RT_{\text{path}}$) separately in the abstract space across the subjects. $S_{1P}$, $S_{1C}$, $S_{2P}$, $S_{2C}$, and $S_{3P}$ indicate the five different abstract spaces.

**Table S2.** The number of navigational paths in the exploration and exploitation stages separated by the *k*-means algorithm according to the learning level predicted by the trained deep neural network (DNN). The *t*- and *p*-values were calculated from the comparison between the number of paths in the exploration and exploitation stages.

| | **Exploration** | **Exploitation** | ***SD*** | ***t*-value** | ***p*-value** |
|---|---|---|---|---|---|
| Overall | 24.84 | 25.16 | 3.53 | -0.23 | .823 |
| 1D | 9.56 | 10.44 | 2.38 | -0.92 | .365 |
| 2D | 10.24 | 9.76 | 1.69 | 0.71 | .485 |
| 3D | 5.04 | 4.96 | 1.62 | 0.12 | .903 |

**Table S3.** Response accuracy ($RA_{path}$) and response time ($RT_{path}$) for each path in the navigation task for the five different abstract spaces, averaged across the subjects ($S_{1P}$, $S_{1C}$, $S_{2P}$, $S_{2C}$, and $S_{3P}$ indicate five abstract spaces; path01, path02, …, path10 indicate ten different paths; *SD* indicates standard deviation).

| | | Average value (*SD*) | | | | | | | | | |
|---|---|---|---|---|---|---|---|---|---|---|---|
| **Dimension** | **Task** | **path01** | **path02** | **path03** | **path04** | **path05** | **path06** | **path07** | **path08** | **path09** | **path10** |
| $RA_{path}$ | | | | | | | | | | | |
| 1D | $S_{1P}$ | 0.93 (0.22) | 0.96 (0.20) | 0.97 (0.13) | 0.91 (0.24) | 0.95 (0.20) | 0.99 (0.05) | 0.94 (0.19) | 0.95 (0.21) | 1.00 (0.00) | 1.00 (0.00) |
| | $S_{1C}$ | 0.94 (0.12) | 1.00 (0.00) | 0.96 (0.20) | 1.00 (0.00) | 1.00 (0.00) | 0.99 (0.07) | 1.00 (0.00) | 0.99 (0.05) | 0.99 (0.06) | 0.96 (0.10) |
| 2D | $S_{2C}$ | 0.68 (0.34) | 0.63 (0.34) | 0.72 (0.33) | 0.63 (0.38) | 0.76 (0.30) | 0.78 (0.32) | 0.64 (0.40) | 0.74 (0.27) | 0.74 (0.26) | 0.81 (0.24) |
| | $S_{2P}$ | 0.66 (0.32) | 0.77 (0.24) | 0.78 (0.30) | 0.77 (0.31) | 0.84 (0.26) | 0.93 (0.18) | 0.86 (0.18) | 0.85 (0.28) | 0.85 (0.17) | 0.84 (0.24) |
| 3D | $S_{3P}$ | 0.56 (0.28) | 0.82 (0.21) | 0.78 (0.30) | 0.79 (0.21) | 0.75 (0.31) | 0.80 (0.28) | 0.80 (0.32) | 0.84 (0.24) | 0.75 (0.28) | 0.82 (0.22) |
| $RT_{path}$ (s) | | | | | | | | | | | |
| 1D | $S_{1P}$ | 1.35 (0.68) | 1.06 (0.41) | 0.97 (0.27) | 1.09 (0.44) | 1.13 (0.40) | 0.97 (0.31) | 1.03 (0.37) | 0.95 (0.38) | 0.87 (0.32) | 0.97 (0.68) |
| | $S_{1C}$ | 1.47 (0.64) | 1.18 (0.46) | 1.14 (0.54) | 1.14 (0.44) | 0.97 (0.42) | 1.18 (0.81) | 1.15 (0.72) | 0.98 (0.47) | 0.90 (0.35) | 1.00 (0.43) |
| 2D | $S_{2C}$ | 2.00 (1.10) | 2.41 (1.66) | 1.93 (0.66) | 2.31 (1.12) | 2.13 (0.91) | 2.41 (1.14) | 2.48 (2.02) | 2.32 (1.39) | 2.18 (0.80) | 2.24 (0.91) |
| | $S_{2P}$ | 2.35 (1.57) | 2.64 (1.26) | 2.81 (2.44) | 2.17 (0.82) | 2.74 (1.85) | 2.36 (1.20) | 2.65 (1.23) | 2.49 (1.27) | 2.22 (1.01) | 2.31 (1.48) |
| 3D | $S_{3P}$ | 2.67 (1.25) | 2.50 (1.35) | 2.79 (1.31) | 2.53 (1.44) | 2.82 (1.37) | 2.67 (1.47) | 2.65 (1.69) | 2.63 (1.37) | 2.79 (1.73) | 2.72 (2.14) |

**Supplementary Note 1**

Fig. S3 shows the behavioral experiments on Day 1. The subjects first filled out three questionnaires and then finished five cognitive tests, which were described as follows.

**Demographic information acquisition.** A questionnaire that we developed was used to acquire basic information about the subjects, including gender, age, education states, and financial situation.

**Sense of direction.** The Santa Barbara Sense of Direction Scale (SBSOD) [1] was used to measure the sense of direction of the subjects. The SBSOD is a 15-item self-report questionnaire. Eight of the 15 items were reverse graded. For each subject, the sense of direction score was calculated by summing the score of the 15 items. A higher sense of direction score indicated a better sense of direction.

**Stress perception.** The Perceived Stress Scale (PSS) [2] was used to measure the stressfulness of the situations that the subjects perceived in the past month. The PSS is a 10-item self-report questionnaire. Four of the ten items were reverse graded. For each subject, the PSS score was calculated by summing across all the 10 items. A higher PSS score indicated greater stress.

**Reasoning ability.** The Raven' Advanced Progressive Matrices Test (APM III) [3] was used to measure the abstract reasoning ability and fluid intelligence (fluid IQ) of the subjects. The APM III is a non-verbal ability test containing 12 practice questions and 36 formal questions. Each question was presented in the form of a matrix with a missing location, which the subjects needed to determine. The subjects got 1 score for

each correct answer of the formal questions. A higher APM score indicated better reasoning ability.

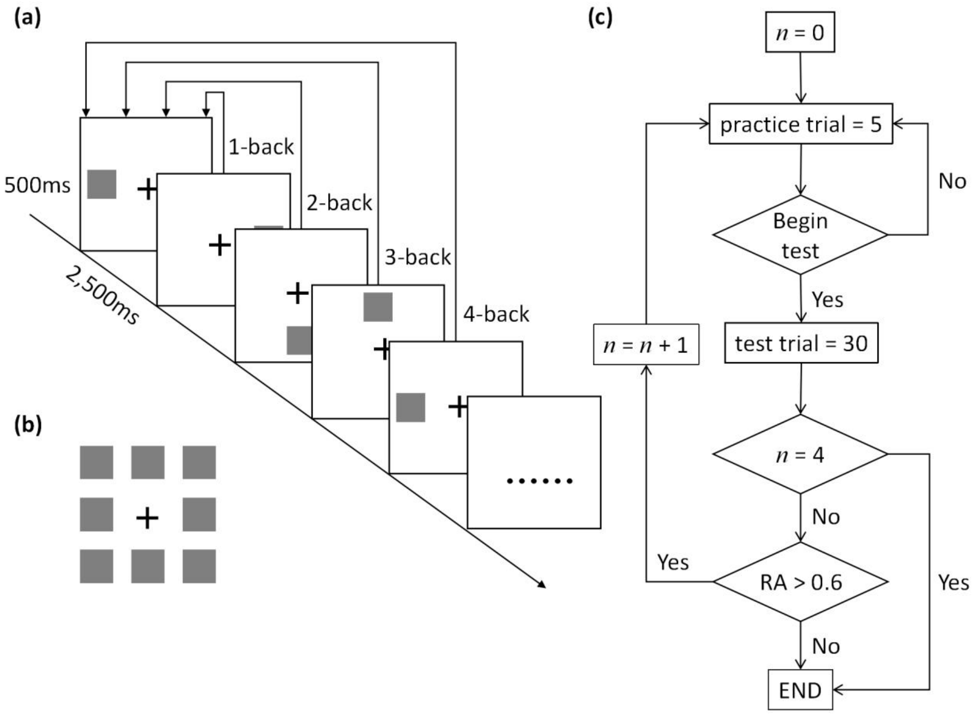


**Fig. S1-1.** Setting and procedure of the spatial *n*-back task used in the experiment on Day 1. **(a)** Procedure of the *n*-back task. In each trial, a square was displayed on the screen for 250 ms and the subjects needed to respond within 2,500 ms before the next trial began. **(b)** The eight potential positions of the square. **(c)** Working memory test. We included four levels of the *n*-back task, $n \in (1, 2, 3, 4)$. For each level of the *n*-back task, the subjects practiced before completing 30 judgements. They were promoted to the next level of *n*-back task if the response accuracy (RA) > 60%. The highest level of the *n*-back task was set to four.

**Working memory.** The spatial *n*-back task [4,5] was used to measure the subjects' working memory. In each trial, a square was presented in one of eight positions (Fig. S1-1b) in the screen for 500 ms. The subjects were asked to judge whether the position of the square was the same as that of the $n^{th}$ trials before within 2,500 ms (Fig.

S1-1a). Four levels of the *n*-back task (1-, 2-, 3-, and 4-back) were used following the procedure shown in Fig. S1-1c. In each level, the subjects first practiced until they thought they were ready and then completed 30 formal judgements. They entered the next level of practice if the response accuracy (RA) was higher than 60%. Otherwise, the task ended.

**Discriminability tests.** Following the procedure of Psychophysics Measure [6], we assessed the subjects' ability to discriminate between the sizes of geometrical shapes (circle or oval), lengths of lines (vertical or horizontal), and the angles between two crossing lines (Fig. S1-2a). In each trial, two stimuli (either two circles, ovals, vertical lines, horizontal lines, or angles) were displayed on the two sides of the screen. The subjects were requested to select the one with larger parameter (size, longer length, or larger angle). The parameter of the left-side stimulus was fixed, whereas the parameter of the right-side stimulus changed according to the following rules. Two sequences, ascending and descending, were set. In the ascending sequence, the size of the right-side stimulus was first set smaller than the left-side stimulus and then increased in each of the following trials until the subjects thought the right stimulus was equal to or larger than the left-side one. In the descending sequence, the size of the right-side stimulus was first set larger than the left-side stimulus and then decreased in each of the following trials until the subjects thought the right-side stimulus was equal to or smaller than the left-side one. Each time after the subjects switched his/her choice, the next sequence began and was set to an ascending or descending sequence randomly. If the subjects successfully judged the size of the

stimuli in a sequence, the amount of change (the change of size in each trial within a sequence) was reduced in the next sequence. The task ended when the subjects failed to discriminate the size of the two stimuli in three continuous sequences. The last stride that the subjects successfully discriminated was recorded as the score of discriminability. A lower score indicated better discriminability.

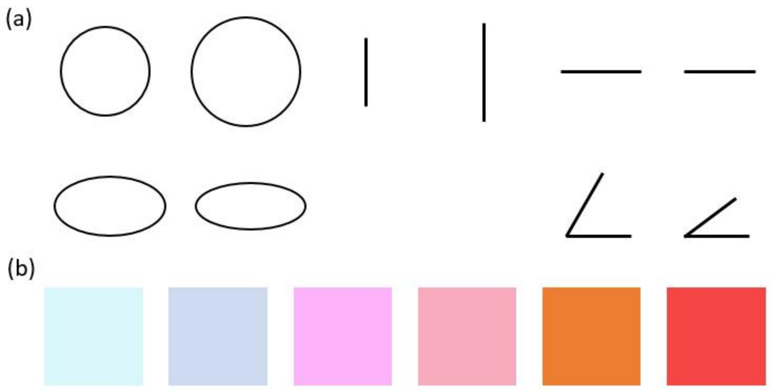


**Fig. S1-2.** Experimental materials for discriminability tests and rule learning used in the experiment on Day 1. **(a)** Materials including circles, ovals, vertical lines, horizontal lines, and angles. **(b)** The color sequence used in rule learning.

**Rule learning.** The rule learning task was used to measure the subjects' ability of learning from feedback. The subjects were instructed to learn the rank of six colors from feedback (Fig. S1-2b). In each trial, two squares with different colors were showed on the screen. The subjects needed to guess and to select the one with the higher rank. Feedback about whether their choice was correct or wrong was displayed on the screen after each selection. When the subjects thought that they had acquired the ranks of the six colors or after they completed 100 trials of learning, they stopped the trials and sorted the colors in sequence.

**Supplementary Note 2**

To ensure that the subjects fully understood the instructions, task procedure, and operations, we requested the subjects to perform the navigation task twice outside the scanner as behavioral training at the end of Day 1 and again just before they entered the MRI scanner on Day 2 (Fig. S3). The two tasks utilized abstract spaces, a 1D space (Fig. S2-1) and a 3D space (Fig. S2-2), that were different from those used in the actual experiment to avoid the subjects becoming familiar with the abstract spaces used in the experimental task-fMRI. These two abstract spaces were entirely different from those used in the task-fMRI experiment. The subjects were asked to perform the navigation task in these two abstract spaces to get familiar with the task interface and procedure. All the subjects used the same abstract spaces during the behavioral training, but the starting points and destinations were shuffled randomly for each subject.

In the behavioral training, feedback on response accuracy (*RA*) and response time (*RT*) was given for each step (Figs. S2-1 and S2-2) as a guide. The subjects were instructed to select the optimal option as quickly as possible. During the navigation task in the 1D space, a green smiling face displayed if the subject chose the optimal option within 1,500 ms. Otherwise (if $RT > 1{,}500$ ms), a sentence displayed to encourage the subject to give faster reactions. When the subject did not choose the optimal option, a red sad face displayed (Fig. S2-1). The feedback in the task of 3D space was same as that in the 1D space, except that the green smiling face displayed when the subject

chose the optimal option within 3,000 ms (Fig. S2-2). In addition, to save time, the interval between the presentation of the destination and the options was narrowed to 2 s, and the fixation cross period between each path and the ITI was fixed at 1 s.

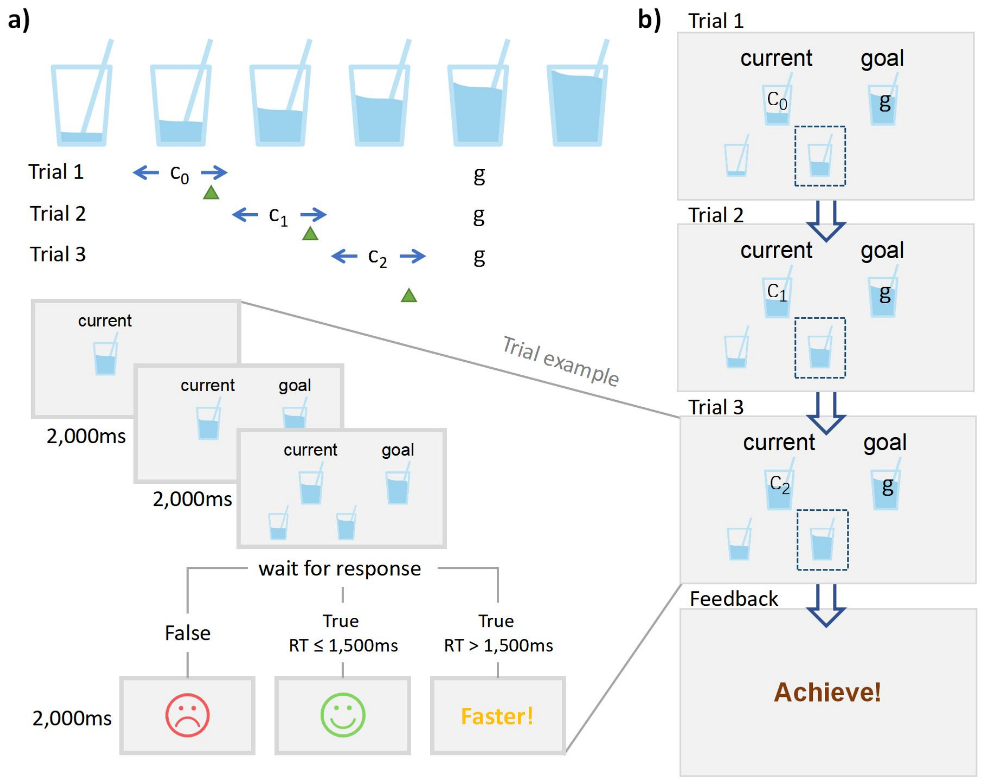


**Fig. S2-1.** Navigation task in the 1D abstract space for the behavioral training. **(a)** The 1D abstract space. **(b)** Procedure of the navigation task. This was same as the procedure shown in Fig. 2a except for the following three settings. First, feedback was given to the subjects after each trial. Second, the interval between the presentation of the destination and the options was narrowed down to 2 s. Third, only two options were provided in each trial. The symbols were created by the authors. Abbreviations: g, navigational goal or destination; $c_n$, the current location of the $n^{th}$ step; RT, response time.

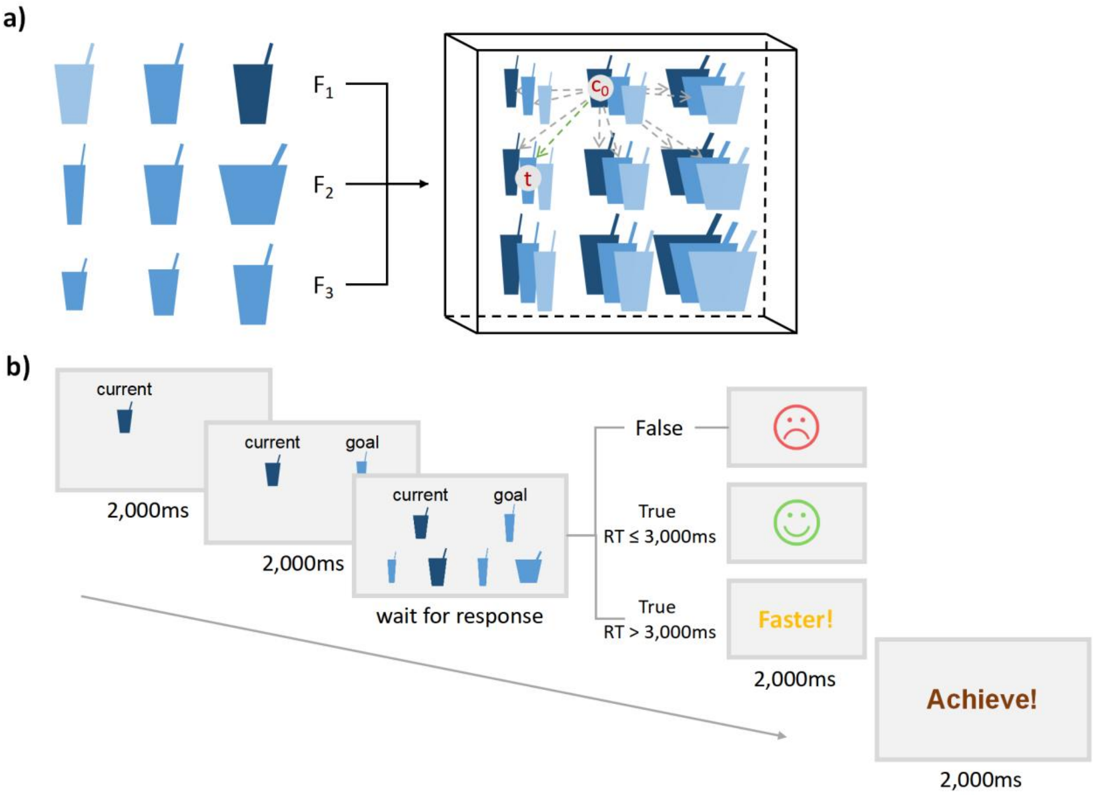


**Fig. S2-2.** Navigation task in the 3D abstract space used in the behavioral training. **(a)** The 3D abstract space. **(b)** Procedure of the navigation task. This was same as the procedure shown in Fig. 2a except for the following two settings. First, feedback was given to the subjects after each trial. Second, the interval between the presentation of the destination and the options was reduced to 2 s. The symbols were created by the authors. Abbreviations: $F_1$, $F_2$, and $F_3$ indicate the three features; g, navigational goal or destination; $c_0$, the current location of the first step; RT, response time.

On Day 1, all the subjects were trained to perform a navigation task in 1D and 3D spaces. First, we described the experimental procedure to the subjects with the assistance of slides. The description was only relevant to the task procedure, including the items displayed on the screen and the operations that the subjects need to perform. The subjects did not receive any information about the structure of the abstract spaces.

Second, the subjects were asked to complete a navigation task including ten paths in the 1D abstract space (Fig. S2-1) and in the 3D space (Fig. S2-2). After the task, we asked the subjects to describe the task procedure and their strategy used in the task. The subjects were asked to repeat the task if they obtained fewer than six goals (obtained less than six coins) in either space. One subject misunderstood the task instruction and repeated the navigation task in the 3D space once. All of the other subjects met the requirement after the first task in the two spaces. In general, the total practice time for each subject was about 4-8 minutes.

On Day 2, the subjects completed a navigation task in the 3D space before the fMRI scanning. After the task, we asked the subjects to describe the task procedure to ensure that they fully understood the task instructions. The subjects were required to complete the task again if they attained fewer than eight goals (i.e., eight coins). All the subjects described the task procedure correctly after the training task. None of the subjects collected fewer than eight coins in this training task. In general, the practice time for each subject was about 3-6 minutes. After the subjects fully understood the experimental procedure and the instructions, we asked them to take a 5-minute short break and then invited them to participate in the subsequent MRI scans.

**Supplementary Note 3**

In the navigation task (both for the behavioral training and the fMRI scanning), at least one optimal location was included in the options, and the other options were selected from the locations surrounding the current location randomly.

*In a 1D space*

Two options were offered in each trial of the navigation task in a 1D space (Fig. S1), because each location was only 'surrounded' by no more than two locations (Fig. 1b). The location to the left of the current location was set as the first option, and the location on the right of the current location was set as the second option. The subjects were instructed to press a button using their left hand to choose the first option and pressing a button using their right hand to choose the second option. When the subjects navigated to the left boundary of the 1D space, a blank image was given as the first option and was set to be non-selectable, because there was no other location on the left side of the current location (Fig. 1b). Similarly, when the subjects navigated to the right boundary of the 1D space, the second option was set to be a blank image and was non-selectable.

*In 2D and 3D spaces*

Four options were offered in the trials of the navigation task in 2D spaces except in the trials in which the subjects navigated to the four corners of a 2D space (surrounded only by three locations) where three options were offered instead (Fig. 2). In each trial, an optimal location was chosen as an option. For example, as shown in

Fig. S7, the subjects can reach the goal location (the circle in orange) with the fewest steps by two possible paths (the yellow and the green lines) from the current location (the circle in blue). Therefore, in this trial, locations 6 and 7 were both optimal locations. First, a location was randomly chosen from the two locations (6 and 7) as the predefined optimal choice. Second, three other locations were chosen from the locations around the current location (within the gray dotted frame). It is possible that both locations 6 and 7 could be chosen as the options. In this situation, whether the subjects chose location 6 or 7, their response was recorded as correct.

The selected options were arranged according to the sequence shown in Fig. S7, with the location below the current location as the first option and the location on the right below the current location as the last option. The other locations were arranged in a clockwise direction. When the subjects navigated to one of the four corners of the 2D space, the current location was only surrounded by three other locations. In this case, the three locations, along with a non-selectable blank image were set as the options. The selection and arrangement of the options in the 3D space was the same as that in the 2D space, except that, when the subjects navigated to the corners of the space, there were still more than four locations for the potential options.

## Supplementary References:


1. Hegarty, M., Richardson, A. E., Montello, D. R., Lovelace, K. & Subbiah, I. Development of a self-report measure of environmental spatial ability. *Intelligence* **30**, 425–447 (2002).
2. Cohen, S., Kamarck, T. & Mermelstein, R. A Global Measure of Perceived Stress. *Journal of Health and Social Behavior* **24**, 385 (1983).
3. John & Raven, J. Raven Progressive Matrices. in *Handbook of Nonverbal Assessment* (ed. McCallum, R. S.) 223–237 (Springer US, Boston, MA, 2003). doi:10.1007/978-1-4615-0153-4_11.
4. Gevins, A. & Cutillo, B. Spatiotemporal dynamics of component processes in human working memory. *Electroencephalography and Clinical Neurophysiology* **87**, 128–143 (1993).
5. Owen, A. M., McMillan, K. M., Laird, A. R. & Bullmore, E. T. N-back working memory paradigm: A meta-analysis of normative functional neuroimaging. *Hum. Brain Mapp.* **25**, 46–59 (2005).
6. Dehaene, S. The neural basis of the Weber-Fechner law: a logarithmic mental number. *TRENDS COGN. SCI.* **7**, 145–147 (2003).
