# Supplementary material for: Forming cognitive maps for abstract spaces: the roles of the human hippocampus and orbitofrontal cortex
Source: Commun Biol. 2024 May 1;7:517. doi: 10.1038/s42003-024-06214-5 (PMC11063219; doi:10.1038/s42003-024-06214-5)
Supplement: Supplementary file 3 — Description of Additional Supplementary Files [file 42003_2024_6214_MOESM3_ESM.pdf]

Description of Additional Supplementary Files

File Name: Supplementary Data 1

Description: Source data used to produce results Figures 2b, 3d, and 3e.

File Name: Supplementary Data 2

Description: Brain regions showing significantly different activations in the exploration and the exploitation stages ($p < .05$, with the cluster forming threshold at $p < .001$).

File Name: Supplementary Data 3

Description: Brain activation significantly associative with the learning level predicted by the deep neural network (DNN). The significance level was set at $p < .05$, with the cluster forming threshold at $p < .001$.

File Name: Supplementary Data 4

Description: Brain activation significantly associative with response accuracy (RA). The clusters are listed in the order of volume size. The significance level was set at $p < .05$, with the cluster forming threshold at $p < .001$.

File Name: Supplementary Data 5

Description: Brain regions with more accurate representation on navigational destination in exploitation than in exploration stage. The clusters are listed in the order of volume size. The significance threshold was set at $p < .01$ (FWE-corrected).
