# Supplementary material for: Forming cognitive maps for abstract spaces: the roles of the human hippocampus and orbitofrontal cortex
Source: Commun Biol. 2024 May 1;7:517. doi: 10.1038/s42003-024-06214-5 (PMC11063219; doi:10.1038/s42003-024-06214-5)
Supplement: Supplementary file 9 — Reporting Summary [file 42003_2024_6214_MOESM9_ESM.pdf]

nature portfolio

Corresponding author(s): DAPR COMMSBIO-23-2026

Last updated by author(s): Jan 14, 2024

Reporting Summary

Nature Portfolio wishes to improve the reproducibility of the work that we publish. This form provides structure for consistency and transparency in reporting. For further information on Nature Portfolio policies, see our Editorial Policies and the Editorial Policy Checklist.

Statistics

For all statistical analyses, confirm that the following items are present in the figure legend, table legend, main text, or Methods section.

n/a	Confirmed	
☐	☒	The exact sample size (*n*) for each experimental group/condition, given as a discrete number and unit of measurement
☐	☒	A statement on whether measurements were taken from distinct samples or whether the same sample was measured repeatedly
☐	☒	The statistical test(s) used AND whether they are one- or two-sided<br>*Only common tests should be described solely by name; describe more complex techniques in the Methods section.*
☐	☒	A description of all covariates tested
☐	☒	A description of any assumptions or corrections, such as tests of normality and adjustment for multiple comparisons
☐	☒	A full description of the statistical parameters including central tendency (e.g. means) or other basic estimates (e.g. regression coefficient) AND variation (e.g. standard deviation) or associated estimates of uncertainty (e.g. confidence intervals)
☐	☒	For null hypothesis testing, the test statistic (e.g. *F*, *t*, *r*) with confidence intervals, effect sizes, degrees of freedom and *P* value noted<br>*Give P values as exact values whenever suitable.*
☒	☐	For Bayesian analysis, information on the choice of priors and Markov chain Monte Carlo settings
☐	☒	For hierarchical and complex designs, identification of the appropriate level for tests and full reporting of outcomes
☒	☐	Estimates of effect sizes (e.g. Cohen's *d*, Pearson's *r*), indicating how they were calculated

Our web collection on statistics for biologists contains articles on many of the points above.

Software and code

Policy information about availability of computer code

Data collection	For data collection, PsychoPy 2021.2.3 was used. The Python codes for running the experiment can be found here: https://www.scidb.cn/s/yy67jq.
Data analysis	Python codes for the machine learning algorithms can be found here: https://www.scidb.cn/s/yy67jq. fMRI Data preprocessing was performed by fMRIPrep 21.0.0 . Univariate and multivariate data analyses were performed by FSL (version 6.0.5.1:57b01774) and Nilearn pakage (https://nilearn.github.io/stable/index.html).

For manuscripts utilizing custom algorithms or software that are central to the research but not yet described in published literature, software must be made available to editors and reviewers. We strongly encourage code deposition in a community repository (e.g. GitHub). See the Nature Portfolio guidelines for submitting code & software for further information.

Data

Policy information about availability of data

All manuscripts must include a data availability statement. This statement should provide the following information, where applicable:

- Accession codes, unique identifiers, or web links for publicly available datasets
- A description of any restrictions on data availability
- For clinical datasets or third party data, please ensure that the statement adheres to our policy

The data that support the findings of this study are available from the corresponding author upon reasonable request.

Research involving human participants, their data, or biological material

Policy information about studies with human participants or human data. See also policy information about sex, gender (identity/presentation), and sexual orientation and race, ethnicity and racism.

Reporting on sex and gender	The gender of each subject was determined based on self-reporting. We analyzed datasets from 12 men and 13 women. To reduce the potential influence of gender differences on the results, we regressed out the variance of gender (and age) in univariate analysis.
Reporting on race, ethnicity, or other socially relevant groupings	All the subjects were recruited from the community of South China Normal University, and all of them are east Asian Chinese people. Therefore, we did not carried out any race or ethnicity based analysis.
Population characteristics	Voluntary subjects had normal or corrected-to-normal vision. None of them had any history of neurological disease or brain disorders.
Recruitment	Participants were recruited using notices on social media and on bulletin boards of researchers.
Ethics oversight	Our study protocol was approved by the Institutional Review Board of South China Normal University (IORG0011738).

Note that full information on the approval of the study protocol must also be provided in the manuscript.

Field-specific reporting

Please select the one below that is the best fit for your research. If you are not sure, read the appropriate sections before making your selection.

☐ Life sciences ☒ Behavioural & social sciences ☐ Ecological, evolutionary & environmental sciences

For a reference copy of the document with all sections, see nature.com/documents/nr-reporting-summary-flat.pdf

Life sciences study design

All studies must disclose on these points even when the disclosure is negative.

Sample size	
Data exclusions	
Replication	
Randomization	
Blinding	

Behavioural & social sciences study design

All studies must disclose on these points even when the disclosure is negative.

Study description	The behavioral performance (accuracy and response time) and the brain response (fMRI) during formal experiment were recorded in quantitative manner.
Research sample	Twenty-seven subjects (14 women) were recruited (mean age, 21.78 years; range, 18-29 years). All had normal or corrected-to-normal vision. None of them had any history of neurological disease or brain disorders.
Sampling strategy	The sample size was determined following previous studies with similar design (Howard et al., 2014, Patai et al., 2019, and Theves et al., 2020).
Data collection	A Siemens Prismafit scanner was used to collect the imaging data. While in the scanner, behavioral choices and response time were recorded using a Python script. Both a technician, blind to the conditions and study hypothesis, and a researcher, not blind to the conditions and study hypothesis were present during the data collections.
Timing	Data were collected from 19/05/2021 until 26/07/2021.
Data exclusions	The data of two subjects were excluded because they did not complete the task.
Non-participation	No participant has dropped out.

Randomization	Participants were not allocated to groups.

Ecological, evolutionary & environmental sciences study design

All studies must disclose on these points even when the disclosure is negative.

Study description	
Research sample	
Sampling strategy	
Data collection	
Timing and spatial scale	
Data exclusions	
Reproducibility	
Randomization	
Blinding	

Did the study involve field work? ☐ Yes ☐ No

Field work, collection and transport

Field conditions	
Location	
Access & import/export	
Disturbance	

Reporting for specific materials, systems and methods

We require information from authors about some types of materials, experimental systems and methods used in many studies. Here, indicate whether each material, system or method listed is relevant to your study. If you are not sure if a list item applies to your research, read the appropriate section before selecting a response.

Materials & experimental systems

n/a	Involved in the study	
☒	☐	Antibodies
☒	☐	Eukaryotic cell lines
☒	☐	Palaeontology and archaeology
☒	☐	Animals and other organisms
☒	☐	Clinical data
☒	☐	Dual use research of concern
☒	☐	Plants

Methods

n/a	Involved in the study	
☒	☐	ChIP-seq
☒	☐	Flow cytometry
☐	☒	MRI-based neuroimaging

Antibodies

Antibodies used	
Validation	

Eukaryotic cell lines

Policy information about cell lines and Sex and Gender in Research

Cell line source(s)

Authentication

Mycoplasma contamination

Commonly misidentified lines
(See ICLAC register)

Palaeontology and Archaeology

Specimen provenance

Specimen deposition

Dating methods

☐ Tick this box to confirm that the raw and calibrated dates are available in the paper or in Supplementary Information.

Ethics oversight

Note that full information on the approval of the study protocol must also be provided in the manuscript.

Animals and other research organisms

Policy information about studies involving animals; ARRIVE guidelines recommended for reporting animal research, and Sex and Gender in Research

Laboratory animals

Wild animals

Reporting on sex

Field-collected samples

Ethics oversight

Note that full information on the approval of the study protocol must also be provided in the manuscript.

Clinical data

Policy information about clinical studies

All manuscripts should comply with the ICMJE guidelines for publication of clinical research and a completed CONSORT checklist must be included with all submissions.

Clinical trial registration

Study protocol

Data collection

Outcomes

Dual use research of concern

Policy information about dual use research of concern

Hazards

Could the accidental, deliberate or reckless misuse of agents or technologies generated in the work, or the application of information presented in the manuscript, pose a threat to:

No | Yes

- [ ] [ ] Public health
- [ ] [ ] National security
- [ ] [ ] Crops and/or livestock
- [ ] [ ] Ecosystems
- [ ] [ ] Any other significant area

Experiments of concern

Does the work involve any of these experiments of concern:

No | Yes

- [ ] [ ] Demonstrate how to render a vaccine ineffective
- [ ] [ ] Confer resistance to therapeutically useful antibiotics or antiviral agents
- [ ] [ ] Enhance the virulence of a pathogen or render a nonpathogen virulent
- [ ] [ ] Increase transmissibility of a pathogen
- [ ] [ ] Alter the host range of a pathogen
- [ ] [ ] Enable evasion of diagnostic/detection modalities
- [ ] [ ] Enable the weaponization of a biological agent or toxin
- [ ] [ ] Any other potentially harmful combination of experiments and agents

Plants

Seed stocks

Novel plant genotypes

Authentication

ChIP-seq

Data deposition

- [ ] Confirm that both raw and final processed data have been deposited in a public database such as GEO.
- [ ] Confirm that you have deposited or provided access to graph files (e.g. BED files) for the called peaks.

Data access links
May remain private before publication.

Files in database submission

Genome browser session
(e.g. UCSC)

Methodology

Replicates

Sequencing depth

Antibodies

Peak calling parameters

Data quality

Software

Flow Cytometry

Plots

Confirm that:

☐ The axis labels state the marker and fluorochrome used (e.g. CD4-FITC).

☐ The axis scales are clearly visible. Include numbers along axes only for bottom left plot of group (a 'group' is an analysis of identical markers).

☐ All plots are contour plots with outliers or pseudocolor plots.

☐ A numerical value for number of cells or percentage (with statistics) is provided.

Methodology

Sample preparation	
Instrument	
Software	
Cell population abundance	
Gating strategy	

☐ Tick this box to confirm that a figure exemplifying the gating strategy is provided in the Supplementary Information.

Magnetic resonance imaging

Experimental design

Design type	Event-related design.
Design specifications	Each participants underwent 5 task-fMRI scanning runs, 2 in 1D abstract space, 2 in 2D abstract space, and 1 in 3D abstract space. Each run of 1D abstract space contained 13-41 steps (mean = 21.56, SD = 5.92); Each run of 2D abstract space contained 19-53 steps (mean = 33.26, SD = 9.26); Each run of 3D abstract space contained 27-52 steps (mean = 37.63, SD = 6.36). The mean time for each trial was 25.08 s, ranging from 11.35 to 84.15 s.
Behavioral performance measures	Number of the trials in each path, the path index, the current location of each trial, the destination of each path, the button pressed, and the response time.

Acquisition

Imaging type(s)	functional and structural images
Field strength	3T
Sequence & imaging parameters	Functional MRI data: single-shot simultaneous multi-slice (SMS) or multi-band (MB) gradient-echo EPI sequence, TR/TE/flip angle = 1,500 ms/31.0 ms/70°, slice acceleration factor = 3 without GRAPPA, FOV = 211 × 211 mm2, data matrix = 88 × 88, slice thickness = 2.4 mm without inter-slice gap, voxel size = (2.4 mm)3, anterior-to-posterior phase encoding direction (A>>P), bandwidth =2,186 hz/px, and 60 interleaved transversal slices (multi-slice mode = interleaved, and series = interleaved) covering the whole brain.<br>Field map: double-echo gradient-echo sequence, TR/TE1/TE2/flip angle = 620 ms/4.92 ms/7.38 ms/60°, FOV = 211 × 211 mm2, voxel size = (2.4 mm)3, and 60 transverse slices.<br>High-resolution brain structural images: T1-weighted 3D MP-RAGE sequence, TR/TE/flip angle = 1,800 ms/2.07 ms/9°, slice thickness = 0.8 mm, FOV = 256 × 256 mm2, data matrix = 320 × 320, voxel size = (0.8 mm)3, and 208 sagittal slices covering the whole brain.
Area of acquisition	Whole brain scans were used.

Diffusion MRI ☐ Used ☒ Not used

Preprocessing

Preprocessing software	The fMRI data were preprocessed using fMRIPrep 21.0.0 with the following steps: (1) generated a reference volume and its skull-stripped brain; (2) estimated the head-motion parameters with respect to the reference volume, including the transformation matrices, and six corresponding rotation and translation parameters; (3) registered the magnetic field coefficients estimated from the field-map to the functional reference volume with rigid-registration; (4) corrected the slice-time of the functional images to 50% of the slice acquisition range (0.702s, 0-1.41s); (5) co-registered the functional reference volume to the T1-weighted 3D images using a boundary-based registration with six degrees of freedom; (6)

calculated the framewise displacement (FD) and extracted the signals within the cerebrospinal fluid (CSF), white matter (WM), and gray matter (GM); (7) resampled the functional data into (2.0 mm)3 in the MNI standard space; (8) used a high pass filter with a cutoff of 1/100 Hz to remove low-frequency drifts. The images were smoothed with an isotropic Gaussian kernel of 6 mm full-width half-maximum (FWHM) for the univariate analysis of the fMRI data. The functional images were not smoothed for the representational similarity analysis (RSA) to retain the multi-voxel pattern

Normalization	Non-linear normalization.
Normalization template	MNI
Noise and artifact removal	We corrected the geometric distortion and signal loss of functional images using field-maps and regressed out 6 rigid body motion parameters during the data analyses. To reduce the impact of excessive head motion on the results, we set the threshold to exclude the data with an average FD > 0.25. No data was excluded according to this threshold. In the statistical analysis, six head-motion parameters estimated using FSL/mcflirt were set as covariates to account for the residual effects of the subjects' movements.
Volume censoring	We did not carry out the volume censoring on our task-fMRI data.

Statistical modeling & inference

Model type and settings	1. Univariate analysis: fixed effects models were used in the 1st and 2nd level analysis, and a random effects model was used in the 3rd or group-level analysis. 2. RSA: the neural RDMs were constructed by calculating the dissimilarity (1-r) between the parameter estimation map of the routes; behavioral RDMs were constructed according to the type of the routes; Spearman correlation was used to assessed the similarity between the neural and behavioral RDMs.
Effect(s) tested	There were two types of trials in the current study, including exploration trials and exploitation trials. These trials were defined by combining a deep neural network and a k-means algorithm.

Specify type of analysis: ☒ Whole brain ☐ ROI-based ☐ Both

Statistic type for inference (See Eklund et al. 2016)	Data are presented including all significant activations at cluster-levels. Local maxima in the clusters were included at peak-level.
Correction	GRF correction voxel p < .001 and cluster p < .05 for the whole-brain univariate analysis (one-sample t-test, two-tailed). FDR correction q < 0.05 for the ROI-based RSA (one-sample permutation test). FWE correction p < 0.05 for the whole-brain searchlight RSA (one-sample permutation with TFCE).

Models & analysis

n/a	Involved in the study
☒	☐ Functional and/or effective connectivity
☒	☐ Graph analysis
☐	☒ Multivariate modeling or predictive analysis

Functional and/or effective connectivity	
Graph analysis	
Multivariate modeling and predictive analysis	Deep neural network (DNN) was used to extract characteristics of behavioral performance (only used on behavioral data but not on the fMRI data). The independent variables included the response accuracy, response time, and steps in each route. Independent data were used to train and test the DNN. We used ReLU as the activation function for the hidden layers and softmax as the activation function for the output layer. Adam optimizer was used to update weights of the network. The learning rate was set to 0.001.
